# Detecting Genetic Variation of Colonizing *Streptococcus agalactiae* Genomes in Humans: A Precision Protocol

**DOI:** 10.3389/fbinf.2022.813599

**Published:** 2022-06-03

**Authors:** Yan Zhou, Xue-Chao Zhao, Lin-Qi Wang, Cheng-Wen Chen, Mei-Hua Hsu, Wan-Ting Liao, Xiao Deng, Qing Yan, Guo-Ping Zhao, Chyi-Liang Chen, Liang Zhang, Cheng-Hsun Chiu

**Affiliations:** ^1^ State Key Laboratory of Genetic Engineering, School of Life Sciences, Fudan University, Shanghai, China; ^2^ Shanghai-MOST Key Laboratory of Health and Disease Genomics, Chinese National Human Genome Center at Shanghai, Shanghai Institute for Biomedical and Pharmaceutical Technologies, Shanghai, China; ^3^ The Institutes of Biology and Medical Sciences, School of Biology and Basic Medical Sciences, Soochow University, Suzhou, China; ^4^ Molecular Infectious Disease Research Center, Chang Gung Memorial Hospital, Chang Gung University College of Medicine, Taoyuan, Taiwan; ^5^ Department of Pediatrics, Chang Gung Memorial Hospital, Chang Gung University College of Medicine, Taoyuan, Taiwan

**Keywords:** group B *Streptococcus*, polymorphic mutation, pooled sample, dominant lineage, colonization

## Abstract

Deciphering the genotypic diversity of within-individual pathogens and verifying the evolutionary model can help elucidate resistant genotypes, virulent subpopulations, and the mechanism of opportunistic pathogenicity. However, observed polymorphic mutations (PMs) are rare and difficult to be detected in the “dominant-lineage” model of bacterial infection due to the low frequency. The four pooled group B *Streptococcus* (GBS) samples were collected from the genital tracts of healthy pregnant women, and the pooled samples and the isogenic controls were genomically sequenced. Using the PMcalling program, we detected the PMs in samples and compared the results between two technical duplicates, GBS-M001T and GBS-M001C. Tested with simulated datasets, the PMcalling program showed high sensitivity especially in low-frequency PMs and reasonable specificity. The genomic sequence data from pooled samples of GBS colonizing carrier pregnant women were analyzed, and few high-frequency PMs and some low-frequency PMs were discovered, indicating a dominant-lineage evolution model. The PMs mainly were nonsynonymous and enriched in quorum sensing, glycolysis/gluconeogenesis, ATP-binding cassette (ABC) transporters, etc., suggesting antimicrobial or environmental selective pressure. The re-analysis of the published *Burkholderia dolosa* data showed a diverse-community model, and only a few low-frequency PMs were shared between different individuals. Genes of general control non-repressible 5-related N-acetyltransferases family, major facilitator superfamily (MFS) transporter, and ABC transporter were positive selection candidates. Our findings indicate an unreported nature of the dominant-lineage model of GBS colonization in healthy women, and a formerly not observed mutation pool in a colonized microbial community, possibly maintained by selection pressure.

## Introduction

The microbiome, which contains a metagenome more than 100-fold of the human genome, may be closely related to the health status of the host (Schwabe and Jobin, 2013; Wirbel et al., 2019). Microbes colonizing at various human body parts such as guts, lungs, stomach, and bladder are suggested to play roles in infection, diabetes, obesity, cardiovascular diseases, and cancers (Tang et al., 2017; Lloyd-Price et al., 2019; Parida and Sharma, 2019; Zhou et al., 2019). The respiratory bacterial communities in cystic fibrosis patients and the host-microbiome interactions may be related to disease status and treatment response, and microbiome modeling could help establish individualized treatment plans and novel therapeutic approaches (Bevivino et al., 2019). Respiratory viral infection-induced microbiome alterations may cause secondary bacterial pneumonia, which often has a more severe clinical course (Hanada et al., 2018).

Metagenome analysis can help understand the different microbial community structures and functions of microbes in distinct individuals (Cai et al., 2017). High-throughput DNA sequencing data has been used to reveal the genomic variety of opportunistic bacteria, which are relevant to the status of individuals. Some opportunistic pathogen species are associated with diseases when there is an outgrowth narrowing the microbe diversity, such as Enterobacteriaceae in intestinal inflammation and infection (Hand 2016). Since only one or several isolates cannot represent the microevolution profile of the species, pooled samples for hundreds of colonies were used in studying the diversity of *Burkholderia dolosa*, an opportunistic pathogen, within cystic fibrosis patients. Six pooled *B. dolosa* samples were sequenced and polymorphic mutations (PMs) with a frequency of more than 0.03 were detected, recording the genomic history of selection on the pathogen within its host (Lieberman et al., 2014). Two evolutionary models of within-patient bacteria were proposed in the study of *Pseudomonas aeruginosa*, another opportunistic respiratory pathogen, the diverse community model and the dominant lineage model (Workentine and Surette, 2011; Chung et al., 2012). In diverse community model, multiple adaptive lineages arise with an intermediate frequency and coexist with other lineages, and most of the PMs found in pooled samples are within an intermediate frequency. In the dominant lineage model, the PMs have much lower frequencies.

Sequencing the genomes of all sampled isolates can yield comprehensive information about a bacterial species, and it has been shown to be suitable for a diverse community model (Marvig et al., 2015; Paterson et al., 2015). However, for a dominant lineage model, this strategy may be ineffective because most isolates are so closely related that there is limited variation among their genomes. On the other hand, genome sequencing of the entire population or the pooled sample is a promising and cost-effective approach for evaluating the diversity of bacterial colonies (Lynch et al*.*, 2014; McAdam et al., 2014). Numerous tools such as MuTect2 (Cibulskis et al., 2013), SNVSniffer (Liu et al., 2016), Lofreq (Wilm et al., 2012), and Strelka2 (Kim et al., 2018) have been developed to detect point mutations or indels (insertions and deletions). For detecting PMs in cancer samples, MuTect2 demonstrates the highest sensitivity. Most PM callers perform well for high frequency (HF) PMs, and for a simulated tumor sample of 90% admixture with a control sequence. The sensitivity of the best low frequency (LF) PM caller, MuTect2, was reported to be 55.2% (Bohnert et al., 2017). The sensitivity of LF-PM detection is important for the dominant lineage model because the frequency of the majority of PMs will be less than 0.1. So, procedures for PM detection with high sensitivity and high accuracy of LF PM are necessarily required, especially for the study of dominant lineage model, in which false positive PMs may be introduced due to sequencing error, contamination, and misalignment.

Group B *Streptococcus* (GBS), also known as *Streptococcus agalactiae*, is the most common cause of neonatal sepsis that is associated with a mortality rate of 5%–20% and serious complications in newborns (Yu et al., 2011). A worldwide review estimates that 1% of all stillbirths in developed countries and 4% in Africa are associated with GBS (Seale et al., 2017). Previous research has often focused on the pathogen invading a host and its interaction with the host defense mechanism (Marvig et al., 2015). However, only a few studies have investigated the long-term effect of bacterial colonization among healthy women. The GBS often colonizes the female genital tract without causing any disease, and it can also infect infants born from colonized women, showing an approximate incidence rate of 50%. The distribution of GBS serotypes in healthy colonized women has been reported to be different from that in infected babies (Wong et al., 2011). The colonization status of GBS in healthy women may help understand the maternal neonate infection and the development of the GBS vaccine.

In this study, an experimental and analytical strategy, with higher sensitivity for PM detection and higher accuracy for filtration has been developed to reach a balance that minimizes the false negatives and false positives, customized for the study of microevolution analysis. After the comprehensive understanding of the metagenome aspect of microbiome community, this method could be utilized to further analyze the recognized key player species, especially the conditional pathogenic bacteria. Using the PMcalling protocol, written in java, we investigated the genomic diversity of GBS, which turned out to be the first genomic example of dominant lineage microevolution model. The validated results may help decipher the diversity of GBS populations, the evolutionary model, and the variations in genes under evolutionary pressure. We also re-analyzed the published data of *B. dolosa* to investigate PMs with lower frequencies, while the reported work mainly focuses on the PMs with higher frequencies (Lieberman et al., 2014).

## Materials and Methods

### Sample Collection

This study was approved by the Institutional Review Board of Chang Gung Memorial Hospital, Taiwan (103-2479B and 104-9360B), and all methods were performed in accordance with the relevant guidelines and regulations. Four samples, GBS-M001, GBS-M006, GBS-M007, and GBS-M008, were collected from the genital tracts of healthy pregnant women enrolled at Chang Gung Memorial Hospital, Taiwan, and all the participants had signed the informed consent. Each sample was cultured in Lim broth (Creative Microbiologicals, Taipei, Taiwan) overnight before being subcultured on a 5% sheep blood agar plate, which was then incubated at 37°C for 24 h. For each sample, 130 random single colonies of GBS were harvested from the plate to construct the pooled sample and transferred to a microcentrifuge tube for DNA extraction. Multiplex PCR was performed using the four pools to test the serotypes of these “isolates” (Wang et al., 2014). Colonies in each pool were confirmed to belong to the same serotype, and the four samples were serotype VI, Ib, Ia, and II, respectively (Supplementary Table S1).

The construction procedure of the isogenic controls was similar to that of the pooled samples. First, a random single colony was picked from 130 colonies of the pooled sample, and then the selected single colony was streaked on a new plate to grow for 24 h. Finally, 130 single clones were randomly selected from the new plate and mixed as the isogenic control sample (Supplementary Figure S1).

### Genome Sequencing

Genomic DNA from the pooled GBS isolates and isogenic controls was extracted using a MoBio UltraClean Microbial DNA Isolation kit (Yu-Shing Biotech, Taipei, Taiwan), and libraries were constructed using an Illumina-compatible Epicentre Nextera DNA Sample Prep kit (NuGen Technologies, San Carlos, CA, United States) according to the manufacturer’s instructions. The entire sequencing was performed on an Illumina MiSeq platform (Illumina Inc., San Diego, CA, United States), yielding paired-end reads (301 nt) for each sample.

Five pooled samples (GBS-M001T and GBS-M001C were technical duplicates of GBS-M001whose genome libraries were constructed and sequenced at Chang Gung Memorial Hospital in Taiwan and the Chinese National Human Genome Center at Shanghai, respectively), four isogenic control samples, and two single clones containing the validated PMs from the pooled GBS-M001 sample were sequenced. The paired-end reads with an overlapping tail sequence were merged *via* Flash (Magoč and Salzberg, 2011) (version 1.2.11). The merged reads were then filtered using sickle (version 1.33) to trim low-quality bases, and reads shorter than 50 bps were discarded.

Two candidate PMs (ref: GBS-M002 70,583 in GBS-M001 G 0.99, A 0.01, and ref: GBS-M002 1,326,769 in GBS-M001 C 0.86, A 0.14) were verified in each single clone of the GBS-M001 pooled sample by PCR. The two pairs of primer were 5ʹ-GCT​TTC​TTG​CCA​TCA​T-3ʹ/5ʹ-TAC​GCA​TCA​AAT​CTG​TTC-3ʹ and 5ʹ-TTC​GCC​AGT​TAC​ATC​AAG-3ʹ/5ʹ-GTC​CGA​GTC​GTG​TCA​GTT-3ʹ. The PCR products were sequenced with ABI3730XL.

### Evaluation of Contamination

The merged paired-end reads were mapped to the Ribosomal Database Project (RDP) database (release11_5_Bacteria_unaligned.fa, http://rdp.cme.msu.edu/misc/resources.jsp) using the BWA (version 0.7.15-r1140) software (Li and Durbin, 2009) and classified into genus using the RDP Classifier (version 2.12) with default settings (Wang et al., 2007).

The selected reads were annotated using BLASTN to the NCBI nt database with default parameters. If the best hit of a read in the BLASTN result was not from GBS, the read was considered a contaminant.

### Proper Reference Selection

A total of 27 complete GBS genomes were downloaded from NCBI. The genomic reads of the GBS pooled samples were aligned to the 27 reference genomes, respectively, using the BWA software. For serotype VI, the reference genomes SG-M8 were added when they were ready online. The SAM tools (version 1.4.1) software (Li et al., 2009) was used to calculate the coverage or the covered reference genome length by three or more reads of the pooled samples over the total length of the reference genome, and the mapping rates, or the number of mapped reads over the total number of high-quality reads. The fixed mutations and the raw PMs of the pooled sample of various reference genomes were obtained using in-house Perl scripts (https://github.com/wanglinqi123/PMcalling) and the PM calling procedure, and then the best reference genome with the highest coverage and mapping rate was selected for further study (Supplementary Table S2).

### Raw Polymorphic Mutations Calling

Unique reads that mapped only one region of the reference genome were retained for PM calling. Find the mismatch and indel sites of each read according to the MD tag and CIGAR value of the SAM file. We get the raw mismatch and fixed mutations when it meets the thresholds (Table 1). After removing the bases near by the end and indel sites, the raw PMs were called from the raw mismatches when the quality thresholds were reached. If there were raw mismatches that meet the fixed threshold, the fixed mutation sites were further supplemented.

**TABLE 1:** PMs found in five GBS samples with PMcalling.

Sample	Freq	Raw PM number	Passed PM number
GBS-M001T	>0.1	494	14
		50	1
	≤0.1	454	13
GBS-M001C	>0.1	465	14
		10	1
	≤0.1	455	13
GBS-M006	>0.1	294	27
		141	2
	≤0.1	153	25
GBS-M007	>0.1	169	16
		7	0
	≤0.1	162	16
GBS-M008		144	53
	>0.1	2	1
	≤0.1	142	52

Freq, frequency.

### Polymorphic Mutations Filtration

Clustered PM filter: If three or more mismatches in a narrow range (50 bps), not counting the fixed mutations, occurred in a single read, then the read was eliminated.

T-test filter: For a PM, the tail distance of every read supporting the major and minor allele was calculated (comparing with the full length of the read) to evaluate bias of the distribution. If the distribution of the tail distance from reads supporting a minor allele was significantly different to that of the major allele in the T-test (*p*-value less than 1E-3), then the minor allele was discarded. In addition, if the distribution of the alignment direction from reads supporting a minor allele was significantly different to that of the major allele in the T-test (*p*-value less than 1E-3), then the minor allele was also discarded.

Isogenic control filter: The isogenic control, a single-colony sample, was used to eliminate false-positive PMs. If a position showed a polymorphism in the isogenic control, the same polymorphism at the same position in the pooled sample was rejected. In addition, if the read number of minor and major allele of the PMs in the pooled sample was not significantly different to that of the same PMs in the isogenic sample in the fisher-test (*p*-value more than 1E-3), then the minor allele was discarded.

Flapping indel filter: If the distance between a PM and a high frequency indel (frequency more than 0.1) was short (for example, 10 bps), then the PM may have been a part of an indel event because of alignment shifting.

### Indel Calling and Filtration

To find the indel sites of each read according to the CIGAR value of the SAM file, we get the raw mismatch (reads> = 4). After removing the bases and indels near by the end, the raw indels were called from the raw mismatch when the quality thresholds (Minread> = 4, MinFreq> = 0.005) were reached. The Isogenic control filter and T-test were same as the PM filtration. A raw indel was discarded if it was caused by homopolymer error (Mardis 2008) that was a single base insertion or deletion and the reference was a string of consecutive bases. The remaining candidate indels were manually checked using Integrated Genome Viewer (IGV, version 2.3.97) to eliminate alignment errors (Thorvaldsdóttir et al., 2013), and in-house Perl scripts were used to mark potentially false indels caused due to sequencing errors such as homopolymer errors to assist with manual filtration (Mardis 2008).

### Performance Evaluation and Parameter Test of Pipeline by Simulated Data

The genome sequence of *S. agalactiae* strain SG-M8 was used as template, and ten mutant sequences with 100 random variant sites were generated with Mutate DNA (http://www.bioinformatics.org/sms2/mutate_dna.html), an online tool of Sequence Manipulation Suite (Stothard 2000). The eleven GBS sequences and the genome of *E. faecalis* strain H25 were treated with ART (Huang et al., 2012), a sequencing read simulator, and twelve paired-end sequencing files in fastq format (Illumina, 250 bp*2) with the depth of 950X were generated. The simulated dataset, admixture.fastq, was composed with 1% of H25.fastq (as contamination), 25% of SG-M8.fastq, and 1%, 2%, 3%, 4%, 5%, 6%, 8%, 10%, 15%, and 20% of MutGBS1.fastq to MutGBS10.fastq respectively, and contained 100 PMs for each of the ten frequencies. GBS-M001 was selected as reference genome, and SG-M8.fastq was used as isogenic control. Another six simulated datasets generated with the same procedure were also used in parameter test.

The numbers of true positive (TP) and false positive (FP) PMs in test, and the total positive PMs (sum of true positive and false negative PMs, TP + FN), were used to evaluate the effectiveness. The sensitivity, aka true positive rate, was calculated as TP / (TP + FN), the precision, aka positive predictive value, was calculated as TP/(TP + FP), and the false positive rate was converted into the number of false positive PMs per million base pairs to indicate the specificity (Cibulskis et al., 2013).

### Pathway Enrichment

**FIGURE 1:**
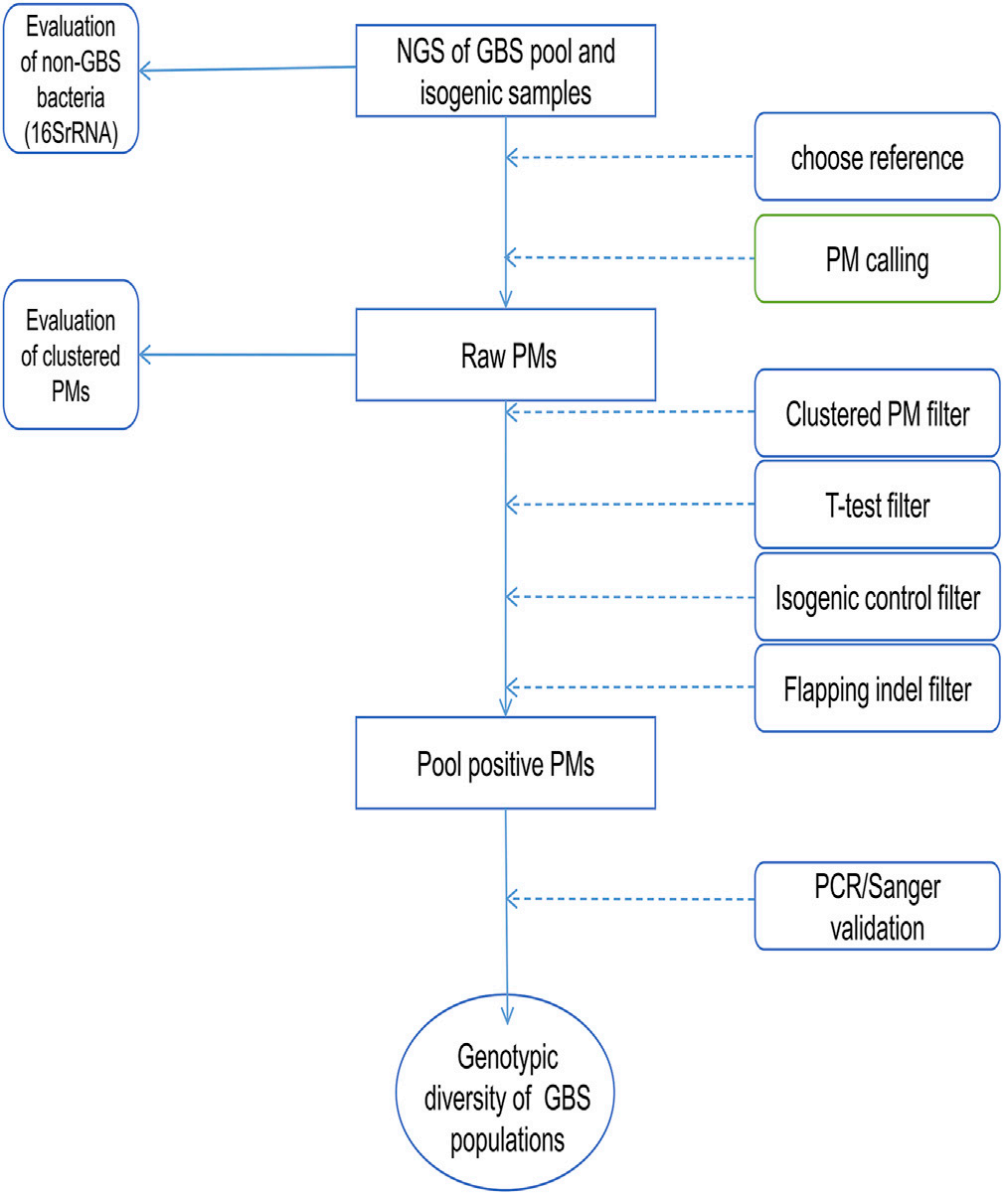
The workflow chart of PMcalling. The next-generation sequencing (NGS) reads of the pooled samples and the isogenic control samples were mapped to the appropriate reference genomes, and the raw PMs were called from the two BAM files by PMcalling. A series of filters were used to remove the false positive PMs introduced by systematic bias, contamination, misalignment, and duplicate regions. Then VCF files of positive PMs and indels, as well as nucleotide sequences of the regions where PMs are located were generated from pooled samples and the isogenic control samples, respectively. PMs validated by PCR/Sanger can be further used for genotypic diversity analysis of GBS.

The candidate positive PMs returned from the PM calling process were further analyzed to identify the genes carrying these PMs and the nonsynonymous PMs. KEGG Pathway analysis and enrichment were performed on genes carrying the candidate positive PMs using an online tool (Xie et al., 2011) (KOBAS 3.0, kobas.cbi.pku.edu.cn). The workflow of this study is shown in Figure 1.

### Positive Select Genes

The expected nonsynonymous mutation ratio of a gene was the average nonsynonymous mutation ratio of all its codens (Supplementary Table S3). Ka/Ks was calculated from the actual Ka/Ks ratio (nonsynonymous PM number/synonymous PM number) divided by the expected Ka/Ks ratio of the gene. Fisher exact test is performed between the actual and the expected nonsynonymous and synonymous PM numbers of the gene.

## Results

### Workflow of Polymorphic MutationsCalling

**FIGURE 2:**
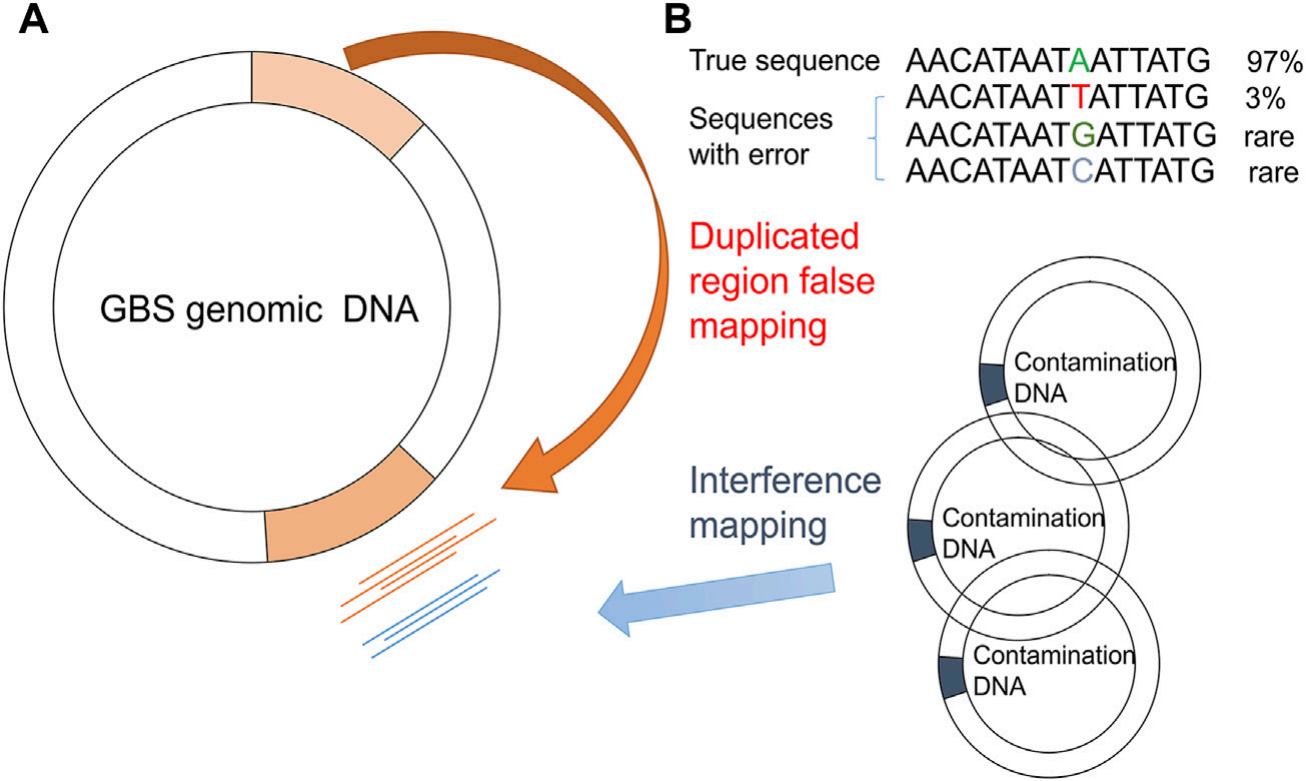
False PM caused by duplicated region/contamination/systematic error (bias). Mapping biases due to GBS genome duplication regions **(A)** and sequencing errors/contaminated DNA **(B)** are shown.

After mapping the NGS reads of the pooled sample and the isogenic control to the reference genome, the PMcalling protocol calls the raw PMs from the two BAM files. Since sequencing error, contamination, and duplicate genomic regions may cause misalignment of short reads and thus generate false positive raw PMs (Figure 2), a series of filters are used (Figure 1). The clustered PM filter will eliminate the sequence reads if more or equal mismatches, not counting the fixed PM, then the parameter MisMatch within a range defined by parameter MisLen is found in a single read. The T-test filter will determine the tail distance, and the alignment direction of each read supporting the major and minor allele. If the distribution of the tail distances supporting a minor allele is significantly different to that of the major allele in a T-test (*p*-value less than 1E-3), or if the distribution of the alignment direction from reads supporting a minor allele is significantly different to that of the major allele in the T-test (*p*-value less than 1E-3), then the minor allele, and the related PM, is discarded. The isogenic control is the negative control, which is samples derived from a single-colony (Supplementary Figure S1). If a position shows a polymorphism in the isogenic control, the same polymorphism at the same position in the pooled sample is rejected. In addition, although the site is not considered a PM in isogenic control (for instance, the minor allele read number is less than the threshold), if the read number of minor and major allele of the PM in the pooled sample is not significantly different to that of the same position in the isogenic sample in the fisher-test (*p*-value more than 1E-3), and then the minor allele will be discarded. The accurate indel calling has been a challenge because of the limited guidelines, low concordance rate among sequencing platforms, alignment error, and incomplete reference genome in some cases (Kumaran et al., 2019), so that the SNVs beside an indel are usually questionable. If the distance between a PM and a high frequency indel (frequency more than 0.1) is shorter than the parameter (indel_distance, 10 bps), then the PM may have been a part of an indel event because of alignment shifting. The Flapping indel filter will remove the related PMs.

### Robustness Test of the Filters With Simulated Data

To test the influence of parameters in PMcalling, a series of simulated GBS sequencing datasets were generated from an isogenic control dataset with artificial PMs of different frequencies. After comparing the sensitivity, positive predictive value, and the false positive rate under different parameters (Supplementary Figures S2A‒S2J), a default combination was selected and used in subsequent analysis (Supplementary Table S4). During the adjustment of some parameters, including “MLength,” “MQuality,” “PMDep,” “FixDep,” and “FixFreq,” the PM numbers found were almost constant. And for the other parameters, such as “MinRead,” “MinFreq,” “End,” and “*p*-value”, LF-PM results are usually slightly affected below allele frequency of 0.02. The combination of “MisMatch” and “MisLen” were the most sensitive parameters, which was effective in the Clustered PM filter. While more positive PMs were found in the results, the number of false positive PMs might increase a little bit. So the parameter values with high sensitivity and an acceptable false positive rate were preferred.

**FIGURE 3:**
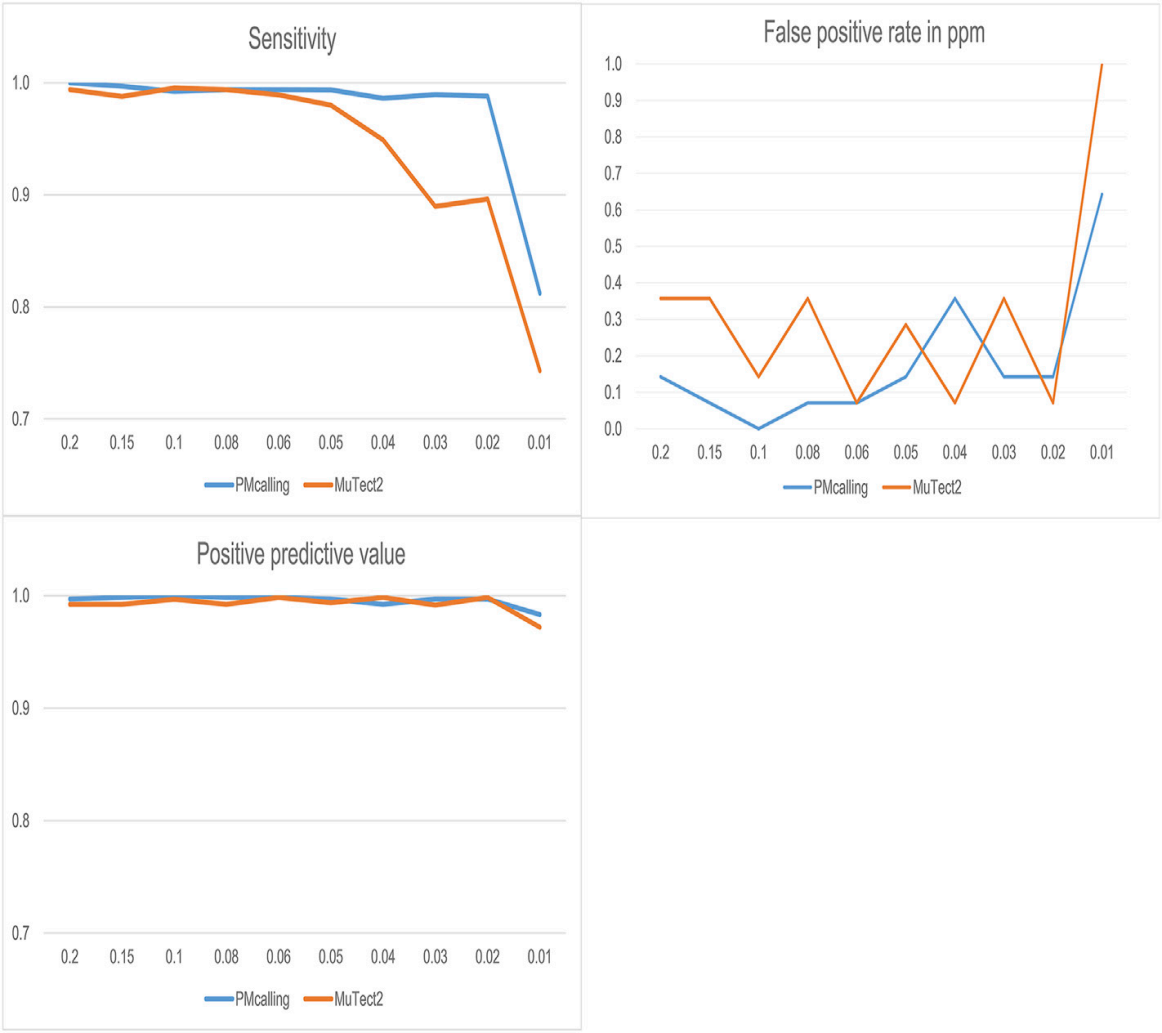
Comparison of PM detection between PMcalling and Mutect2 with simulated datasets. The sensitivity is calculated by dividing the positive PM number found in the result by the total number of positive PM in simulated datasets for each frequency. The Positive predictive value is calculated by dividing the positive PM number found in the result by the total number of PM (positive and false positive) found for each frequency. The false positive rate is converted into the false positive PM number per million basepairs found in simulated datasets for each frequency.

PMcalling and MuTect2 were used for PM detection in simulated datasets, and both protocols showed good specificity, and PMcalling had higher sensitivity for the LF-PMs (Figure 3). For the PMs with frequency more than 0.05, the two methods both could find more than 98% of the positive PMs, and for the PMs with frequency 0.02–0.04, PMcalling detected 98% of the positive PMs while MuTect2 detected 90%. For the PMs with frequency 0.01, the sensitivities of PMcalling and MuTect2 decreased to 81% and 74%.

Considering that PMs may have frequencies less than 0.01, we constructed a simulated dataset containing PMs with frequency 0.005 (removing PMs with frequency 0.2) in a similar way and detected PMs with Lofreq, Mutect2, PMcalling, SNVSniffer, and Strelka2 (Supplementary Table S5; Supplementary Figure S3). For the PMs with frequency more than 0.05, all the five variant callers could detect more than 93% of the positive PMs. The sensitivity of SNVSniffer and Lofreq gradually decreased when the PMs frequency was between 0.02 and 0.04. While PMcalling, Mutect2, and Strelk2 performed steadily and were able to detect more than 90% of the positive PMs. For the PMs with frequency 0.005, the sensitivity of PMcalling, Mutect2, and Strelk2 decreased to 46%, 23%, and 5%, and the positive predicted value decreased to 81%, 61%, and 38%, with false positive rate of 6.2, 4.7, and 3.8 per million base pairs, respectively.

### Validation of Polymorphic Mutations Detection in Technical Duplicates of Group B *Streptococcus*

Two datasets of the pooled sample GBS-M001, GBS-M001T and GBS-M001C are technical duplicates, which were sequenced twice from individual sequencing libraries, constructed from the same sample DNA. These two duplicates were used for PM detection case study between PMcalling and MuTect2.

**FIGURE 4:**
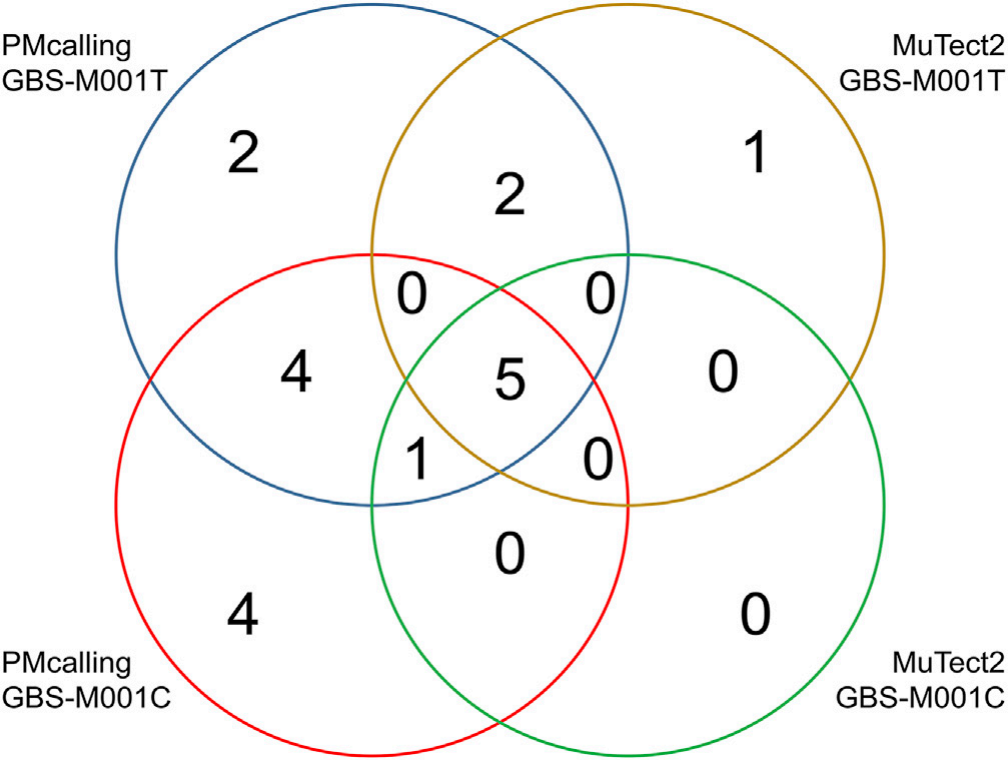
Number of PMs found in technical duplicates with PMcalling and MuTect2.

Among the total 18 GBS-M001 PMs, 14 (78%) were found in GBS-M001C with a sequencing depth of >900x, and 14 (78%) were found in GBS-M001T with a sequencing depth of >700x. Ten PMs including the only HF-PM (ref: GBS-M002 1,326,769, A 0.14/C 0.86) was shared in both samples. This indicates a reasonable sensitivity for LF-PMs, which could be estimated at 71% (10/14). There were 8 PMs found by MuTect2 in GBS-M001T, and seven of them were shared with those found by PMcalling. All the 6 PMs found by MuTect2 in GBS-M001C were included in PMcalling results (Supplementary Table S6). Five MuTect2 detected PMs were shared between GBS-M001T and GBS-M001C. The PMcalling has almost doubled sensitivity for finding more potential PMs, comparing to MuTect2, with the same proportion of shared PMs in technical duplicates (Figure 4).

### Polymorphic Mutations Detection in Group B *Streptococcus* Pooled Samples

Besides the two duplicate samples, GBS-M001T and GBS-M001C, a total of five GBS pooled samples were analyzed. The PMs found in the samples are from 14 to 53, and there were only 0 to 2 HF-PMs in each sample (Table 1). Most of the discarded raw PMs, from 77% to 97%, were trimmed by “Clustered filter.” The fact that rare HF-PMs existed in GBS suggested a dominant lineage evolution model of the samples.

### Nonsynonymous Positive Polymorphic Mutations in the Coding Area

Of the 18 positive PMs found in the two GBS-M001 pooled samples, 13 PMs were in the coding regions of 13 genes, of which 11 (85%) were nonsynonymous PMs. The other three samples, GBS-M006, GBS-M007, and GBS-M008, resulted in similar trends, wherein more than half of the PMs in the coding regions (58%–91%) were nonsynonymous mutations. These mutations may have influenced the function of 71 genes in total (Supplementary Table S7). Pathway analysis (Supplementary Table S8) demonstrated that these genes were enriched (*p*-value less than 0.05) in Quorum sensing, Glycolysis/Gluconeogenesis, ABC transporters, Biosynthesis of secondary metabolites, Pyrimidine metabolism, Degradation of aromatic compounds, Purine metabolism, Biosynthesis of antibiotics, and Nicotinate and nicotinamide metabolism. In addition, the verified LF-PM in GBS-M001 was located in the aldehyde-alcohol dehydrogenase gene (WP_000137036.1, p.G504S), and the LF-PM belonged to the iron-containing alcohol dehydrogenase (Fe-ADH) domain (Pfam: PF00465, 470-858). The verified HF-PM in GBS-M001 was located in the bifunctional pyrimidine operon transcriptional regulator (WP_000823056.1, pyrR, p.S168I).

### Polymorphic Mutations Detection of Published *B. Dolosa* Datasets

Six public genomic datasets of pooled *B. dolosa* samples were analyzed with PMcalling process. There were 112, 63, 79, 84, 54, and 257 PMs with an allele frequency more than 0.03 found in six samples, respectively (Supplementary Table S9). The 0.03 allele frequency was the threshold used by the original publication to eliminate error prone LF-PMs. Among the total 616 nonredundant PMs, 468 (76%) PMs were shared with the reported result which included 678 PMs (Lieberman et al., 2014), while 252 (94%) HF-PMs (frequency more than 0.1) were shared (Supplementary Figure S4). When the frequency threshold was set to more than 0.02, there were 1,830 nonredundant PMs found in six samples (Supplementary Table S10), and 1,393 PMs of them were in coding regions in 1,109 genes, including 1,061 nonsynonymous mutations and 332 synonymous mutations (Table 2). A total of 22,410 PMs with a frequency more than 0.01 were detected (Supplementary Table S3), and 17,634 PMs were in coding regions of 4,395 genes including 13,215 nonsynonymous mutations and 4,419 synonymous mutations.

**TABLE 2:** PMs, synonymous and non-synonymous mutations found in six *B. dolosa* samples with PMcalling at different frequency thresholds.

Freq threshold	PM number	Synonymous mutation number	Non-synonymous mutation number
>0.02	1830	332	1061
>0.01	22410	4419	13215

Freq, frequency.

We found 53 genes potentially under positive selection (Supplementary Table S11) with eight or more nonsynonymous PMs (allele frequency more than 0.01, and Ka/Ks ratio more than 2). Three genes annotated as general control non-repressible 5-related N-acetyltransferase (GNAT) family, DUF839 domain-containing protein, and major facilitator superfamily (MFS) transporters were found significantly different from the expected distribution of nonsynonymous and synonymous PM numbers by Fisher exact test (*p*-value less than 0.05), suggesting positive selection. All the 15, 13, and 11 PMs in the three genes were nonsynonymous.

## Discussion

Since the theoretical frequency of PM in pooled sample may be as low as 1%, the thresholds of the parameters are widened to include more raw PMs and increase the sensitivity. The strict filters are designed to eliminate the false PMs that introduced by sequencing error, contamination, misalignment, and duplicate genomic regions, so that it can reach a balance between sensitivity and specificity. When the allele frequency was at a high level, the adjustments of most protocol parameters influenced the PM results slightly when using simulated data, so that the robustness is reasonable in the high frequency region. However, the variation of some parameters showed significant effects on the detection of LF-PMs when the allele frequency was below 0.02, with the combination of “MisMatch” and “MisLen” being the most sensitive parameters. Because false positives would be easily eliminated compared with false negatives in further experimental validation, the balance toward sensitivity of PMcalling is acceptable.

The validation using simulated sequencing data showed that PMcalling performed higher sensitivity and predictive positive value for detecting the LF-PMs compared with the other four variant callers. The possible reason is that the commonly used variant callers, such as Mutect2, Lofreq, and Strelka2 are designed mainly for detecting somatic variants in the human genome. Compared to the bacterial genome, the human genome is diploid and has many repetitive regions. The complexity of the human genome may increase the difficulty of variants detection, and also limit the threshold of filtering parameters for many variant callers. PMcalling has been customized for detecting variants, or PMs, from the genomic data of pooled bacterium samples. The filtering parameters of PMcalling are tailored to specific models and therefore may be more rigorous and effective in targeting bacterial genomic variation. However, real data are much more complicated, and the sensitivity and specificity may be lower than simulated data.

The proposed advantage of PMcalling in variant detection of pooled bacterium samples was further described in an analysis of two GBS technical duplication samples, with experimentally validated PMs. The PMcalling has almost doubled sensitivity for finding more potential PMs, comparing to Mutect2, with the same proportion of shared PMs in technical duplicates. PMcalling and Mutect2 shared Five PMs in the T and C duplicates, and one of them, the HF-PM (ref: GBS-M002 1,326,769, A 0.14/C 0.86), was validated (Supplementary Table S6). However, a validated LF-PM (ref: GBS-M002 70,583, A 0.01/G 0.99) was shared in both samples in PMcalling results but was absent in MuTect2 results. The validation of this PM added credits to the sensitivity of PMcalling. A MuTect2 detected raw LF-PM (ref: GBS-M002 1,912,682, C 0.01/T 0.99) was filtered as “clustered” in sample GBS-M001T, but was a PASS in sample C. A PM (ref: GBS-M002 593,534, C 0.01/T 0.99) was found only in GBS-M001T by MuTect2 and was marked as “clustered PM” by PMcalling. The according reads were aligned to known bacterium genomes, finding that they were contaminations from *Enterococcus*. Another PM (ref: GBS-M002 1,750,144, C 0.01/T 0.99) was found in both samples with PMcalling, and was discarded as “alt_allele_in_normal,” which means found in isogenic control by MuTect2 in GBS-M001T. Further validation is needed. Two shared and six unique PMs in C and T samples, were detected by PMcalling but not by Mutect2. MuTect2 ignored these PMs mainly because of their low frequency. Since the pooled sample is composed of 130 strains, the frequency of a single variant could be less than 0.01. After checking the IGV files of the LF-PMs manually, we retained these PMs for further verification. Two PMs (ref: GBS-M002 396,424, T 0.01/C 0.99, and ref: GBS-M002 1,320,879, A 0.01/T 0.99) were found in GBS-M001T by both PMcalling and MuTect2, but were absent in GBS-M001C. These PMs indicate the possibility of missing LF-PMs in a single experiment by chance. If taking the “sensitivity of sample” into consideration, duplicate samples would be a better sequencing strategy for dominant lineage models, contributing to a higher sensitivity of PM detection. Two random sampling processes are more efficient than double the depth of single sequencing experiment. If the sensitivity of a single analysis is about 60%–70%, the duplicates may potentially increase it to 80%–90%.

Choosing a proper reference genome helps eliminate false positives caused by duplicate genomic region errors. For example, there was a one-copy region in GBS strain A, whereas there were two copies of this region in strain B. When we used A as a reference, there was a B-like strain in our pooled sample, and the NGS reads of the two regions may be mapped onto the only region present in strain A. False-positive PMs and HF-PMs may occur in bases that were different between the two genomic regions during the PM calling processes. We found that the coverage rate of the reference genome and the mapping rate of the sample reads were relevant. In addition, the advantage of choosing a reference with a high coverage and mapping rate was to lower the number of fixed mutations, which would interfere with proper read mapping if there were too many. Therefore, the reference genomes with the highest coverage and mapping rates and the lowest number of fixed mutations were selected for further analysis.

With improvements in genome sequencing technology, longer reads provide more information than previously obtained to improve the accuracy of the haplotype, or clustered PM, detection, which has been proven to be essential to filter out duplicated regions and contamination causing false-positive results in this study. Most of the raw PMs were discarded in the clustered PM filter, and contamination and duplicated genomic regions of some strains in the pooled sample resulted in all of the clustered PMs. Interestingly, reads of the HF clustered PMs were often mapped to two or more adjacent duplicate regions of the SG-M8 genome, which was sequenced by the PacBio platform. The PacBio platform provides long reads to distinguish repeat regions in the genome. It may be difficult to assemble these adjacent duplicate regions correctly in genomes with relatively short reads sequenced by the Illumina platform. However, due to the existence of horizontally transferred genes and highly conserved regions in bacteria, there is a small risk that a positive PM is considered a contaminant, and the accuracy of this filtration method needs further verification. The isogenic control was generated from a single colony randomly selected from the pooled sample, and its genomic reads should theoretically not have had any positive PMs, so the PMs found in isogenic control were assumed to be introduced from systematic bias. Only 8–20 variants were found in the four isogenic control samples, and the total size of GBS genome was more than 2 million base pairs, so there was little probability that a true PM was accidentally identical with the variant found in the isogenic control. In this study, most of the false-positive PMs were discarded in the clustered PM filter, and the isogenic control filter appeared to have a limited effect, except that approximately half of the raw indels were eliminated by this filter when the clustered filter was not efficient after merging adjacent indels, due to random alignment shifts around the indel region. The other half of the raw indels were considered to be possible homopolymer errors, which often occurred when there were six or more identical bases in a row (Mardis, 2008). When there was a long string of identical nucleotides in the genome, a false indel of the same base may have been introduced. Homopolymer errors were one of the primary disadvantages of the 454 sequencing platform and may also occur in other platforms at a much lower frequency (Marinier et al., 2015).

Using the validated PMcalling protocol, we reported a dominant lineage evolution model of GBS carrier pregnant women using a pooled sample sequencing strategy. In particular, detection of LF-PMs has been improved. Previously published studies have primarily focused on the diverse community model supported by HF-PM observation algorithms (Boitard et al., 2012; Ferretti et al., 2013; Lieberman et al., 2014). Sequencing the genomes of every bacterial isolate collected from a single individual is a straightforward strategy for a diverse community model. However, in a dominant lineage model, this strategy may not be effective because of the homogeneity among individual genomes. Genome sequencing of the entire population or pooled isolates is a promising approach despite the technical challenges of identifying LF-PMs. Taken together, the average sequencing depths of the pooled and isogenic samples in this study ranged from 486 to 1,048 (Supplementary Table S1), and the positional depths calculated using the number of reads mapped to each base pair position of their best reference genomes ranged from 400 to 900, which ensured the sensitivity of the LF-PMs.

A large portion of the positive PMs in the coding regions was nonsynonymous, suggesting positive selection pressure in those affected genes. Interestingly, some of these genes were found repeatedly in different samples (Supplementary Table S7), and these genes were related to ABC transporter, dihydrofolate reductase, phosphoribosylformylglycinamidine synthase, and family stress response membrane protein. Dihydrofolic acid (conjugate base dihydrofolate) is a folic acid (vitamin B9) derivative that is converted into tetrahydrofolic acid by dihydrofolate reductase. Tetrahydrofolate is required to produce both purines and pyrimidines, which are the building blocks of DNA and RNA. The dihydrofolate reductase has been targeted by various drugs to prevent nucleic acid synthesis (Podnecky et al., 2017). The pathway analysis showed enrichments of genes with nonsynonymous PMs in some vital pathways, including quorum sensing, glycolysis/gluconeogenesis, pyrimidine and purine metabolism. Quorum sensing is involved in the development of resistance to multiple drugs in the microbe (Chen et al., 2016). Extended samples and further investigations may help elucidate how the diversity of GBS and the frequency of certain PMs change over time or under different environmental cues.

This study was inspired by the work of Lieberman et al. (2014), who used pooled samples to investigate microevolution of *B. dolosa* within patients, and found it was consistent with the diverse community evolution model. A total of 678 PMs (allele frequency more than 0.03) were reported. The PMcalling was used to re-analysis these public genomic data of pooled bacterial samples and focused on LF-PMs. The result of public *B. dolosa* datasets showed a large number of LF-PMs, and the PM number increased from 616 to 1,830 and 22,410 while the frequency threshold decreased from 0.03 to 0.02 and 0.01. Interestingly, only a few (1%–3%) LF-PMs were shared between each two samples from different individuals, with allele frequency threshold from 0.03, 0.02 to 0.01. This finding suggests that in each *B. dolosa* colonized microbial community, there is a formerly not observed mutation pool, which probably contributes to the response to the environment or host stress. Three genes annotated as GNAT family N-acetyltransferase, DUF839 domain-containing protein, and MFS transporter, were good positive selection candidates, and the functions of the former two genes were both unclear and needed further study. The major facilitator superfamily (MFS) was one of the two largest families of membrane transporters, and some members of MFS associated with antimicrobial-resistant were found under positive selection in *Salmonella* (Liao et al., 2019). Besides the genes related to MFS transporter and ABC transporter, the other candidate genes (Supplementary Table S11) have been reported to be under positive selection in bacteria, such as porin (Vuotto et al., 2017), DNA topoisomerase (Rojas et al., 2017), OmpA family protein (Ngwamidiba et al., 2006), glutamine--fructose-6-phosphate transaminase (Liao et al., 2019), cytochrome P450 (Wan et al., 2017), and penicillin-binding protein 2 (Zhan and Zhu, 2018). The research on three penicillin-binding proteins of *Streptococcus pneumoniae* identifies several sites which are positively selected and correlated with discriminating amoxicillin MIC values (Stanhope et al., 2008). Evidence of positive selection in a penicillin-binding protein 2a of methicillin-resistant *Staphylococcus aureus* suggests the selective pressure operating the gene is not only related to the antibiotic use, but is more probably related to the host’s inflammatory or immune response during infection (Zhan and Zhu, 2018). Some virulence factor genes of *Streptococcus pyogenes* are also found interacting with human immune system and face direct selective pressures (Wilkening and Federle, 2017). The mechanism of copious *B. dolosa* strains with major LF-PMs colonizing in host may be associated with the effect of very long-time interaction with host immune system, and selection pressures may maintain these LF-PMs.

## Conclusion

We have provided a new tool to investigate the genomic data of microevolution study, digging into the low frequency variation field, which has not been systematically analyzed before. Considering the influence of subtle contamination, duplicated regions, and sequencing platform errors, a protocol of LF-PM calling has been suggested. After the comprehensive understanding of the metagenome aspect of microbiome community, this protocol could be utilized to further analyze the recognized key player species (Jordán et al., 2015), especially the conditional pathogenic bacteria. Our data are the first genomic-level description of the population structure of a “dominant lineage” bacterial evolution model given the fact that rare HF-PMs existed in GBS samples among healthy people. Although the dominant strains of GBS were susceptible to antibiotics, minor strains carried mutations in some vital pathways, which may be the result of antimicrobial or environmental selective pressure. This finding has been supported by the re-analysis of the published genomic data of *B. dolosa* samples with diverse community evolution model. The results of this study will help decipher the diversity of GBS colonization and variations in genes under evolutionary pressure have implications in the development of GBS vaccine.

## Data Availability

The datasets presented in this study can be found in online repositories. The name of the repository and accession number can be found below: NCBI; PRJNA428783.
