# Supplementary material for: Detecting Genetic Variation of Colonizing Streptococcus agalactiae Genomes in Humans: A Precision Protocol
Source: Front Bioinform. 2022 Jun 3;2:813599. doi: 10.3389/fbinf.2022.813599 (PMC9580942; doi:10.3389/fbinf.2022.813599)
Supplement: Supplementary file 2 [file DataSheet4.DOCX]

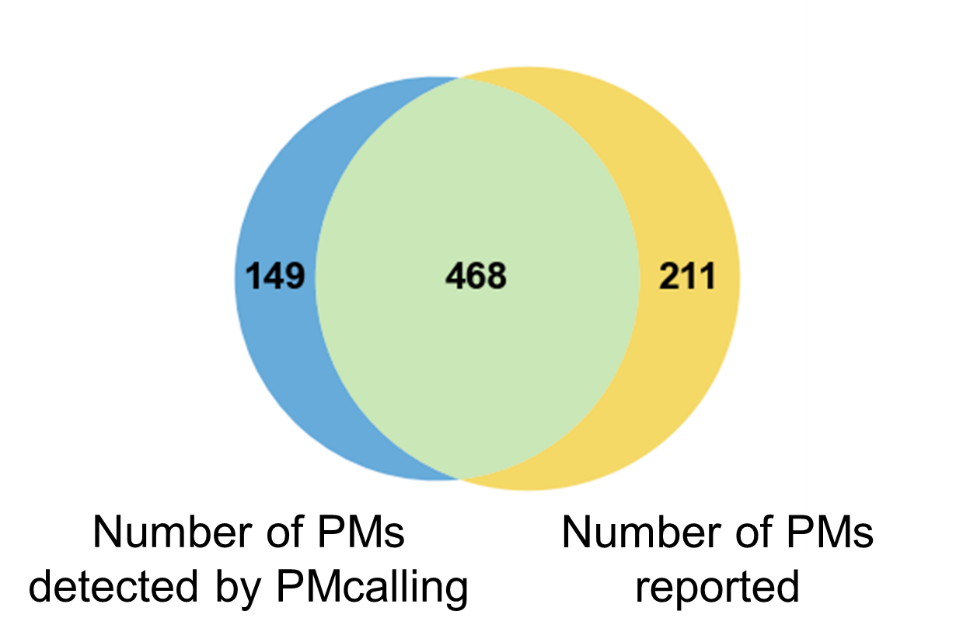


**SUPPLEMENTARY FIGURE 4 |** Venn diagram of PMs detected by PMcalling and reported in the six public *Burkholderia dolosa* genomic datasets when the frequency threshold was 0.03. Among the total 616 nonredundant PMs, 468 (76%) PMs were shared with the reported result which included 678 PMs.
