# Supplementary material for: Detecting Genetic Variation of Colonizing Streptococcus agalactiae Genomes in Humans: A Precision Protocol
Source: Front Bioinform. 2022 Jun 3;2:813599. doi: 10.3389/fbinf.2022.813599 (PMC9580942; doi:10.3389/fbinf.2022.813599)
Supplement: Supplementary file 5 [file DataSheet3.DOCX]

**SUPPLEMENTARY FIGURE 3 | Comparison of PM detection among PMcalling and the other four variant callers with simulated datasets.** The sensitivity is calculated by dividing the positive PM number found in the result by the total number of positive PM in simulated datasets for each frequency. The Positive predictive value is calculated by dividing the positive PM number found in the result by the total number of PM (positive and false positive) found for each frequency. The false positive rate is converted into the false positive PM number per million basepairs found in simulated datasets for each frequency.
