# Supplementary material for: Detecting Genetic Variation of Colonizing Streptococcus agalactiae Genomes in Humans: A Precision Protocol
Source: Front Bioinform. 2022 Jun 3;2:813599. doi: 10.3389/fbinf.2022.813599 (PMC9580942; doi:10.3389/fbinf.2022.813599)
Supplement: Supplementary file 10 [file DataSheet2.DOCX]

**SUPPLEMENTARY FIGURE 2A | Parameter adjustment: MLength.** The sensitivity is calculated by dividing the positive PM number found in the result by the total number of positive PM in simulated datasets for each frequency. The Positive predictive value is calculated by dividing the positive PM number found in the result by the total number of PM (positive and false positive) found for each frequency. The false positive rate is converted into the false positive PM number per million basepairs found in simulated datasets for each frequency.

**SUPPLEMENTARY FIGURE 2B | Parameter adjustment: Mquality.** The sensitivity is calculated by dividing the positive PM number found in the result by the total number of positive PM in simulated datasets for each frequency. The Positive predictive value is calculated by dividing the positive PM number found in the result by the total number of PM (positive and false positive) found for each frequency. The false positive rate is converted into the false positive PM number per million basepairs found in simulated datasets for each frequency.

**SUPPLEMENTARY FIGURE 2C | Parameter adjustment: MinRead.** The sensitivity is calculated by dividing the positive PM number found in the result by the total number of positive PM in simulated datasets for each frequency. The Positive predictive value is calculated by dividing the positive PM number found in the result by the total number of PM (positive and false positive) found for each frequency. The false positive rate is converted into the false positive PM number per million basepairs found in simulated datasets for each frequency.

**SUPPLEMENTARY FIGURE 2D | Parameter adjustment: MinFreq.** The sensitivity is calculated by dividing the positive PM number found in the result by the total number of positive PM in simulated datasets for each frequency. The Positive predictive value is calculated by dividing the positive PM number found in the result by the total number of PM (positive and false positive) found for each frequency. The false positive rate is converted into the false positive PM number per million basepairs found in simulated datasets for each frequency.

**SUPPLEMENTARY FIGURE 2E | Parameter adjustment: End.** The sensitivity is calculated by dividing the positive PM number found in the result by the total number of positive PM in simulated datasets for each frequency. The Positive predictive value is calculated by dividing the positive PM number found in the result by the total number of PM (positive and false positive) found for each frequency. The false positive rate is converted into the false positive PM number per million basepairs found in simulated datasets for each frequency.

**SUPPLEMENTARY FIGURE 2F | Parameter adjustment: PMDep.** The sensitivity is calculated by dividing the positive PM number found in the result by the total number of positive PM in simulated datasets for each frequency.The Positive predictive value is calculated by dividing the positive PM number found in the result by the total number of PM (positive and false positive) found for each frequency.The false positive rate is converted into the false positive PM number per million basepairs found in simulated datasets for each frequency.

**SUPPLEMENTARY FIGURE 2G | Parameter adjustment: FixDep.** The sensitivity is calculated by dividing the positive PM number found in the result by the total number of positive PM in simulated datasets for each frequency. The Positive predictive value is calculated by dividing the positive PM number found in the result by the total number of PM (positive and false positive) found for each frequency. The false positive rate is converted into the false positive PM number per million basepairs found in simulated datasets for each frequency.

**SUPPLEMENTARY FIGURE 2H | Parameter adjustment: FixFreq.** The sensitivity is calculated by dividing the positive PM number found in the result by the total number of positive PM in simulated datasets for each frequency. The Positive predictive value is calculated by dividing the positive PM number found in the result by the total number of PM (positive and false positive) found for each frequency. The false positive rate is converted into the false positive PM number per million basepairs found in simulated datasets for each frequency.

**SUPPLEMENTARY FIGURE 2I | Parameter adjustment: MisMatch & MisLen.** The sensitivity is calculated by dividing the positive PM number found in the result by the total number of positive PM in simulated datasets for each frequency. The Positive predictive value is calculated by dividing the positive PM number found in the result by the total number of PM (positive and false positive) found for each frequency. The false positive rate is converted into the false positive PM number per million basepairs found in simulated datasets for each frequency.

**SUPPLEMENTARY FIGURE 2J | Parameter adjustment: P-value.** The sensitivity is calculated by dividing the positive PM number found in the result by the total number of positive PM in simulated datasets for each frequency. The Positive predictive value is calculated by dividing the positive PM number found in the result by the total number of PM (positive and false positive) found for each frequency. The false positive rate is converted into the false positive PM number per million basepairs found in simulated datasets for each frequency.
