# Supplementary material for: Detecting Genetic Variation of Colonizing Streptococcus agalactiae Genomes in Humans: A Precision Protocol
Source: Front Bioinform. 2022 Jun 3;2:813599. doi: 10.3389/fbinf.2022.813599 (PMC9580942; doi:10.3389/fbinf.2022.813599)
Supplement: Supplementary file 15 [file DataSheet1.DOCX]

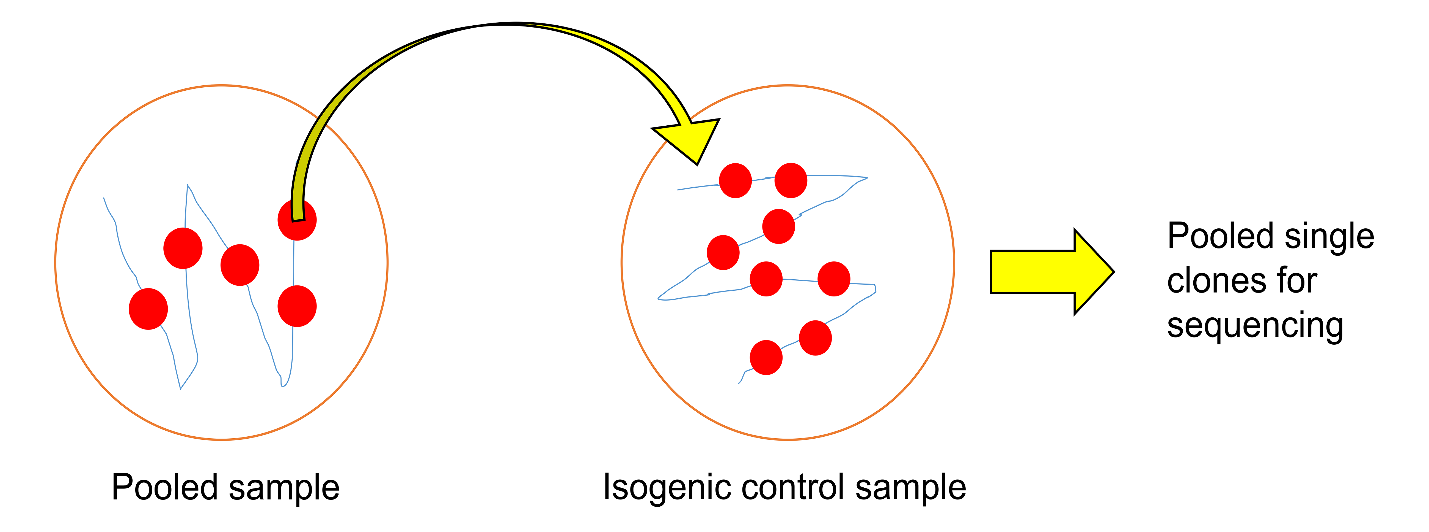


**SUPPLEMENTARY FIGURE 1 |** Schematic diagram to demonstrate collection of an isogenic control for analysis of polymorphic mutations.
